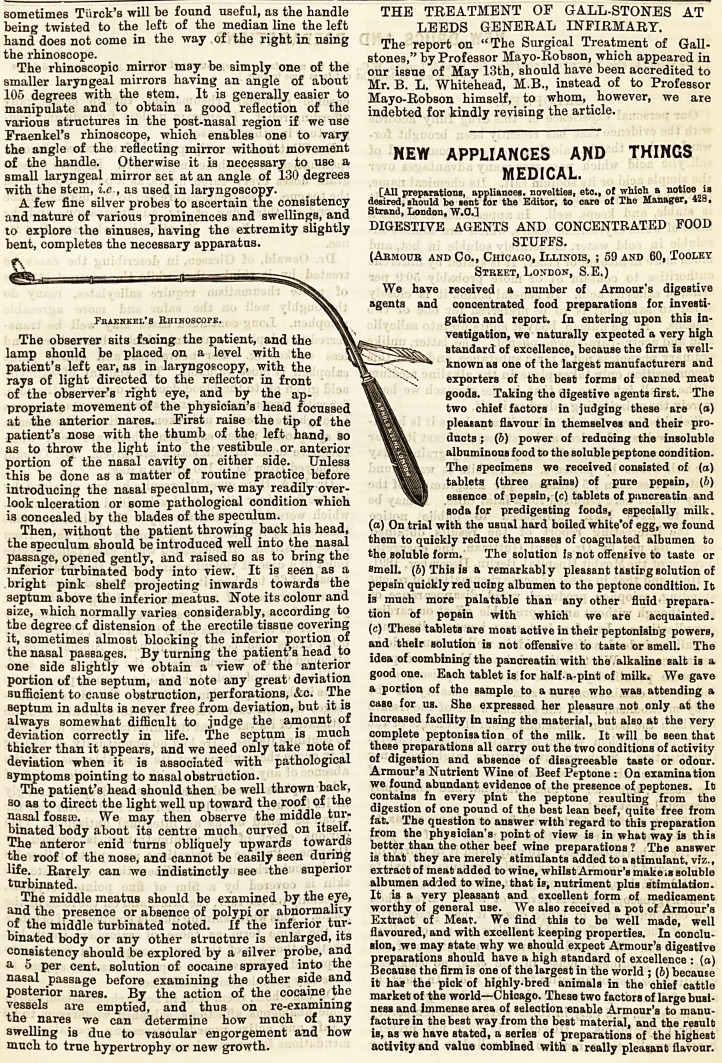# Rhinoscopy

**Published:** 1893-05-27

**Authors:** P. Watson Williams

**Affiliations:** Physician to the Throat Department, Bristol Royal Infirmary


### RHINOSCOPY.—III.

### Examination of the Nose.

P. Watson Williams, M.D.Lond., Physician to the Throat Department, Bristol Royal Infirmary.

For a satisfactory examination of the nose and nasopharynx a brilliant light is essential. Artificial light is nearly always employed, as sunlight, although an excellent illuminant, requires careful management, for posterior rhinoscopy at any rate, and, unless special reflectors are used, the collected rays may burn the parts.

We do not propose to give a complete description of the very numerous forms of illuminating apparatus, but

Fig. 1.—Hurst's portable accumulators for the electric light and cautery. The current is generated by connecting the wires, and regulated by simply rotating the rheostat wheels.

a brief allusion to some of the more commonly employed lamps may be helpful to our readers. Of these, certainly the most convenient and cleanly is Morell-Mackenzie's gas bracket with funnel and a bull's-eye condenser to collect the rays. It is very essential that the light should be freely moveable in every direction, so as to allow of ready adjustment in focussing the light on the part to be examined. It is usually supplied by an Argand burner, but a very much whiter, purer, and more powerful illumination can be obtained by substituting the 80 candle-power Welsbach incandescent burner. A valuable feature in this Welsbach burner is the adjustment for rotating the light on its axis. The drawback to the Welsbach light is the very fragile incandescent curtain, and unless one is resident in a town where the curtains are stocked it is advisable to keep an extra one at hand in case the one in use becomes destroyed. When available, the oxyhydrogen limelight surpasses every other illuminant in brilliancy and purity; but it is only in particular cases that its advantages outweigh the increased difficulties in adjustment. Its cost too is considerable, and therefore none but specialists would find it worth their while to provide themselves with the apparatus.

It is, however, a great convenience to have a portable light, and for many practical reasons that will be obvious to practitioners, the electric light is to be recommended. Portable accumulators, which illuminate

Fraenkel's. Lennox Browne's. Duplay's.

Nasal Specula.

a small electric lamp for several hours in the aggregate, are made by most manufacturing electricians, and for portability and simplicity we can strongly commend Hurst's, which weighs about 14 lb. The smaller electric lamps have up till recently been rather unsatisfactory, but Dr. Washington Isaac has introduced a lamp with a parabolic reflector, so that the reflected rays of the lamp in his focus are all parallel. As these lamps are detachable, and may be used in any direction for illuminating various parts of the body, they are sure to prove useful for many purposes. In any but the larger towns, however, the difficulties in getting the accumulators recharged will prevent many medical men availing themselves of this excellent lighting apparatus.

The reflecting mirror is the same as that used in laryngoscopy of about 14-inch focus, although one of shorter focus, similar to that for examining the ear, is sometimes recommended. It has an opening in the centre through which the eye of the observer looks.

It is more or less a matter of taste whether the mirror is fixed to the forehead by means of a band round the head or a metal band over the head, such as Fox's head band, or is carried on a spectacle frame. But it is essential that the central opening should come immediately in front of the observer's corresponding pupil, and that the mirror should be freely adjustable.

Of nasal specula there is an abundant choice Of the most generally useful we may mention Lennox Browne's, consisting of two ivory blades on sliding bars. It may be used in cautery operations as well as for all other purposes. Fraenkel's is very commonly employed, but the fenestra allow the vibrissæ to project and obstruct the view. Duplay's modification of Bresgen's is very comfortable to the patient.

Fraenkel's. Turck's.

Tongue Depressors.

For operations on the nose requiring two hands, some form of speculum, which is readily fixed and retained in position, is desirable.

Of tongue depressors Fraenkel's is very handy, but sometimes Türck's will be found useful, as the handle being twisted to the left of the median line the left hand does not come in the way of the right in using the rhinoscope.

The rhinoscopic mirror may be simply one of the smaller laryngeal mirrors having an angle of about 105 degrees with the stem. It is generally easier to manipulate and to obtain a good reflection of the various structures in the post-nasal region if we use Fraenkel's rhinoscope, which enables one to vary the angle of the reflecting mirror without movement of the handle. Otherwise it is necessary to use a small laryngeal mirror set at an angle of 130 degrees with the stem, *i.e.*, as used in laryngoscopy.

A few fine silver probes to ascertain the consistency and nature of various prominences and swellings, and to explore the sinuses, having the extremity slightly bent, completes the necessary apparatus.

FRAENKEL'S RHINOSCOPE.

The observer sits facing the patient, and the lamp should be placed on a level with the patient's left ear, as in laryngoscopy, with the rays of light directed to the reflector in front of the observer's right eye, and by the appropriate movement of the physician's head focussed at the anterior nares. First raise the tip of the patient's nose with the thumb of the left hand, so as to throw the light into the vestibule or anterior portion of the nasal cavity on either side. Unless this be done as a matter of routine practice before introducing the nasal speculum, we may readily overlook ulceration or some pathological condition which is concealed by the blades of the speculum.

Then, without the patient throwing back his head, the speculum should be introduced well into the nasal passage, opened gently, and raised so as to bring the inferior turbinated body into view. It is seen as a bright pink shelf projecting inwards towards the septum above the inferior meatus. Note its colour and size, which normally varies considerably, according to the degree of distension of the erectile tissue covering it, sometimes almost blocking the inferior portion of the nasal passages. By turning the patient's head to one side slightly we obtain a view of the anterior portion of the septum, and note any great deviation sufficient to cause obstruction, perforations, &c. The septum in adults is never free from deviation, but it is always somewhat difficult to judge the amount of deviation correctly in life. The septum is much thicker than it appears, and we need only take note of deviation when it is associated with pathological symptoms pointing to nasal obstruction.

The patient's head should then be well thrown back, so as to direct the light well up toward the roof of the nasal fossæ. We may then observe the middle turbinated body about its centre much curved on itself. The anterior end turns obliquely upwards towards the roof of the nose, and cannot be easily seen during life. Rarely can we indistinctly see the superior turbinated.

The middle meatus should be examined by the eye, and the presence or absence of polypi or abnormality of the middle turbinated noted. If the inferior turbinated body or any other structure is enlarged, its consistency should be explored by a silver probe, and a 5 per cent. solution of cocaine sprayed into the nasal passage before examining the other side and posterior nares. By the action of the cocaine the vessels are emptied, and thus on re-examining the nares we can determine how much of any swelling is due to vascular engorgement and how much to true hypertrophy or new growth.

## Figures and Tables

**Fig. 1. f1:**
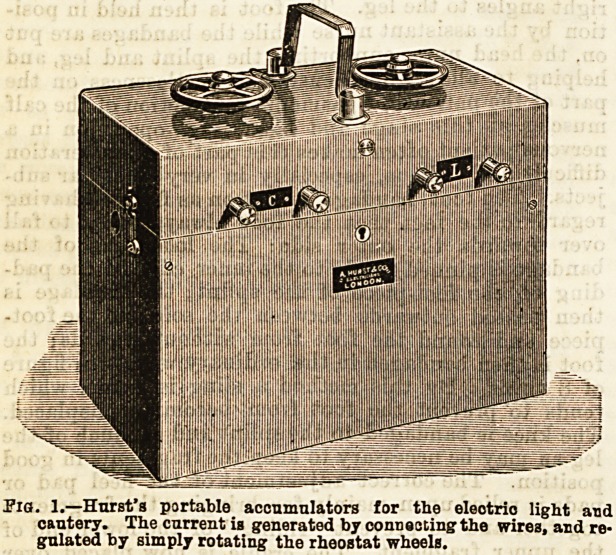


**Figure f2:**
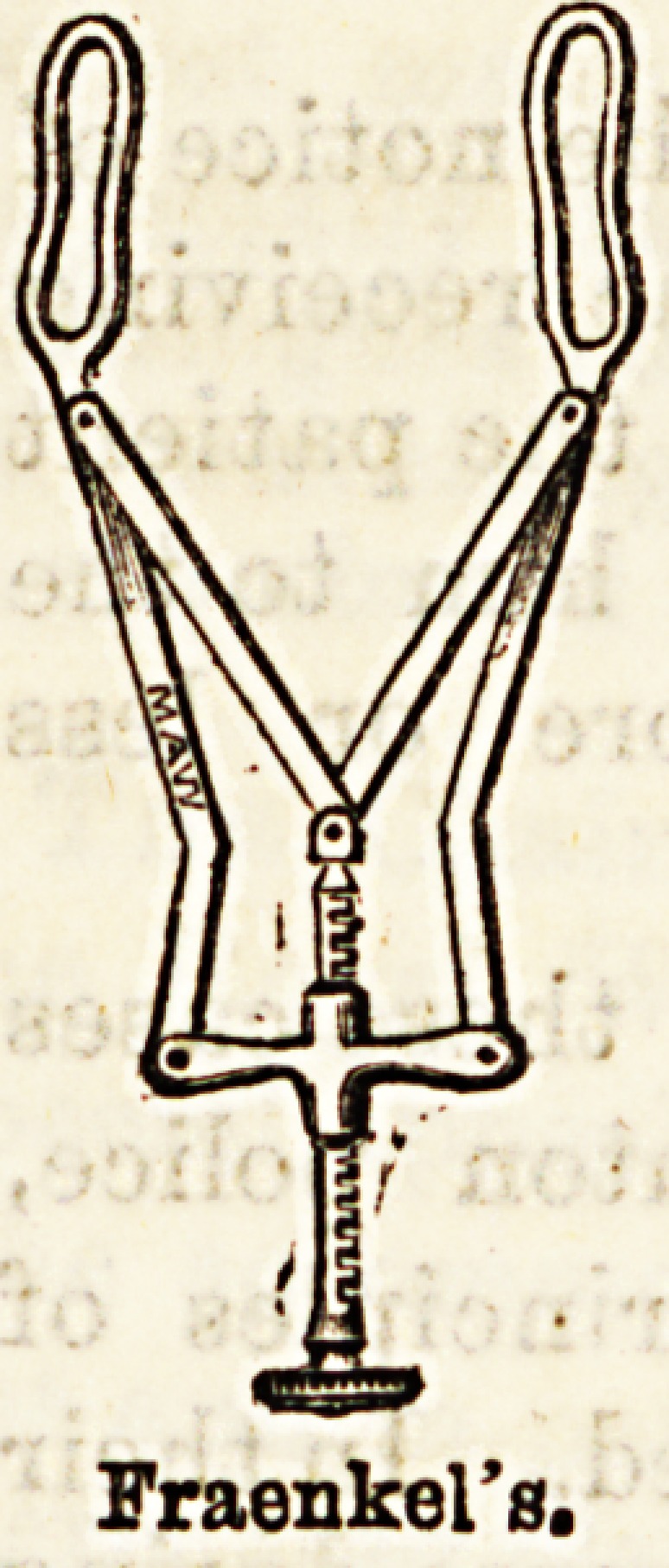


**Figure f3:**
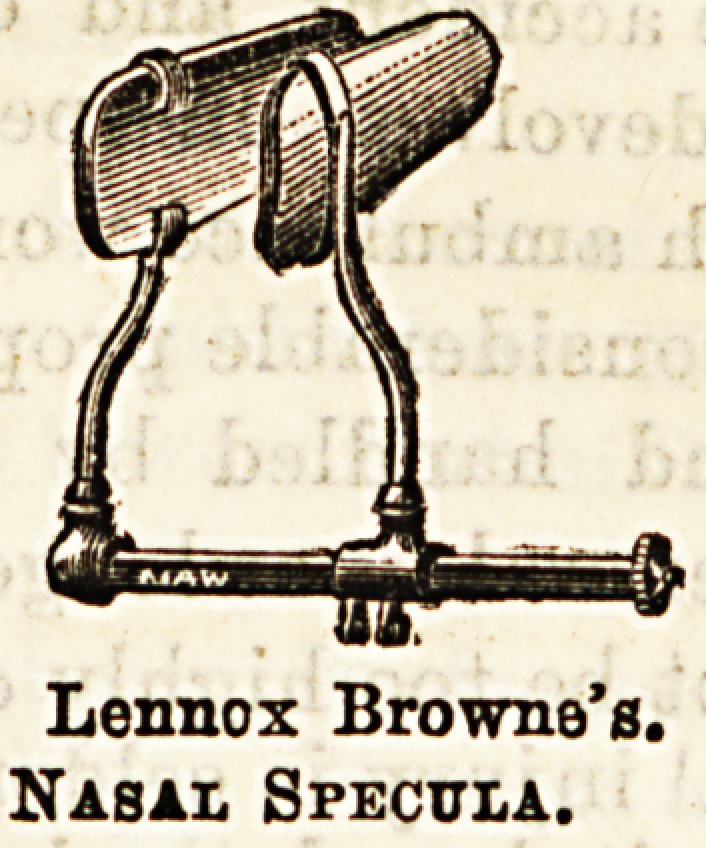


**Figure f4:**
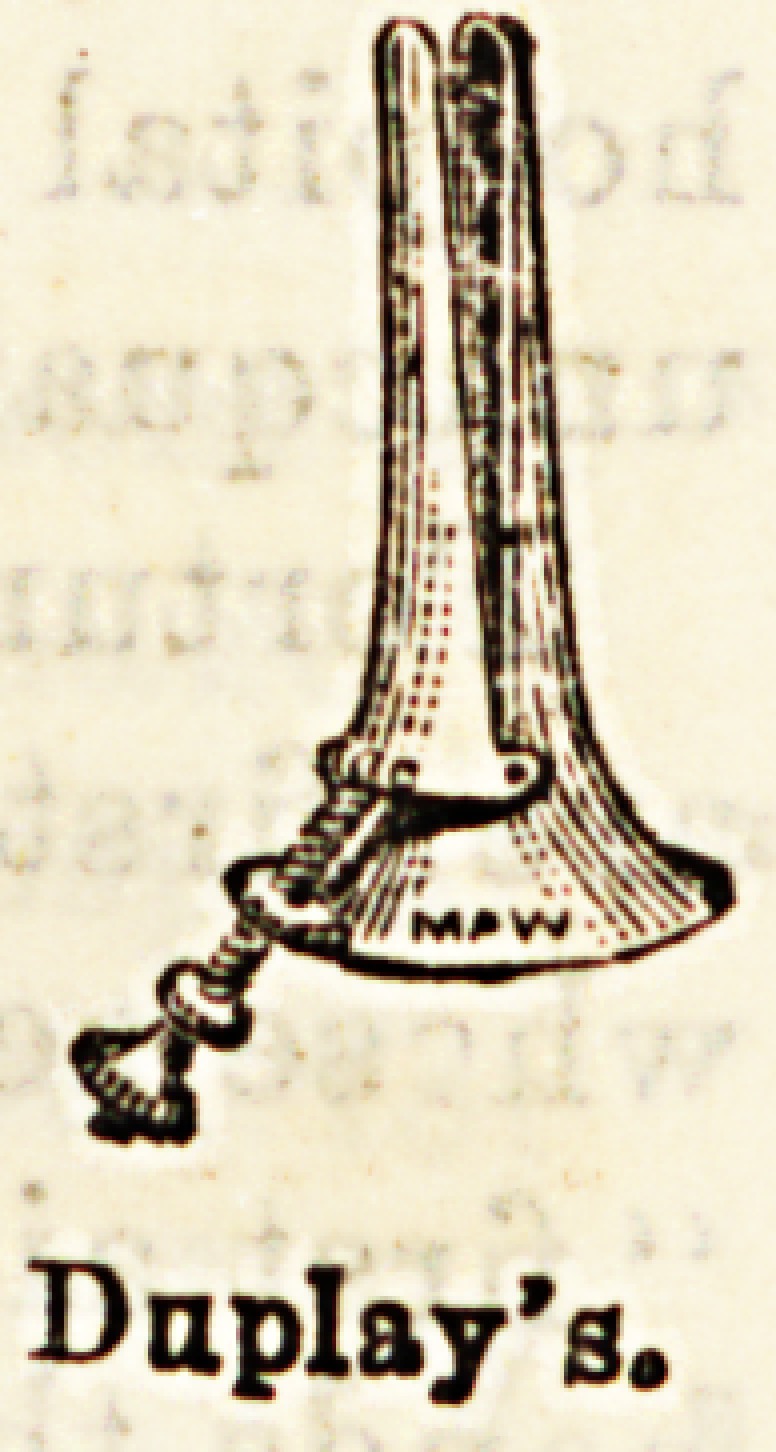


**Figure f5:**
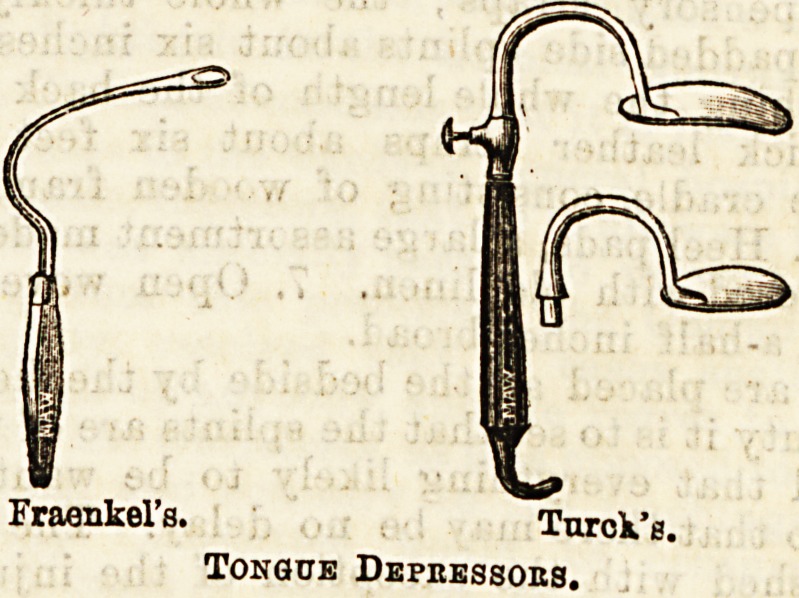


**Figure f6:**